# Electrocardiograph signal denoising based on sparse decomposition

**DOI:** 10.1049/htl.2016.0097

**Published:** 2017-06-29

**Authors:** Junjiang Zhu, Xiaolu Li

**Affiliations:** Mechanical and Electronic Engineering Institute, China Jiliang University, Xueyuan Road 258, Jianggan District, Hangzhou, Zhejiang, People's Republic of China

**Keywords:** electrocardiography, medical signal processing, signal denoising, time-frequency analysis, iterative methods, ECG signal denoising, sparse decomposition, linear denoising method, sparse-based method, myoelectric interference, matching pursuit algorithm, MIT-BIH arrhythmia database

## Abstract

Noise in ECG signals will affect the result of post-processing if left untreated. Since ECG is highly subjective, the linear denoising method with a specific threshold working well on one subject could fail on another. Therefore, in this Letter, sparse-based method, which represents every segment of signal using different linear combinations of atoms from a dictionary, is used to denoise ECG signals, with a view to myoelectric interference existing in ECG signals. Firstly, a denoising model for ECG signals is constructed. Then the model is solved by matching pursuit algorithm. In order to get better results, four kinds of dictionaries are investigated with the ECG signals from MIT-BIH arrhythmia database, compared with wavelet transform (WT)-based method. Signal–noise ratio (SNR) and mean square error (MSE) between estimated signal and original signal are used as indicators to evaluate the performance. The results show that by using the present method, the SNR is higher while the MSE between estimated signal and original signal is smaller.

## 1 Introduction

ECG signals, which are among the most important bio-electricity signals of the human body, reflect the heart's electrical activity and other reliable features from various aspects. Naturally, these signals can serve as vital clinical references for the diagnosis and treatment of cardiological diseases. However, noises are inevitable when ECG signals are being collected. The subsequent results would be tampered if those noises are left untreated. The noises in ECG signals generally come from three aspects: power-line interference, the most common interference, whose frequency is 50 or 60 Hz and whose interference amplitude is able to reach 50% of the peak value of the ECG signals; myoelectric interference, whose noises are generated due to myoelectric contraction and has wide frequency with spectral characteristic similar to that of white noise; baseline interference will cause fluctuation and even distortion of the ECG signal waves with its main energy gathering around 0.1 Hz. Myoelectric interference has difficulty in getting rid of noises due to the asymmetry of the circuit in the data collecting device, although there is a trap circuit to control the noises in most data acquisition circuit.

To solve this problem, many scholars have proposed various methods. Among all the methods, template-based techniques [1, 2] and principle component analysis [3–5] denoise method are often used; however, both methods work only when R peak has been detected in processing single lead ECG. Therefore, filter-based [6, 7] method, empirical mode decomposition [8, 9] and wavelet transform (WT) [10, 11]-based method are developed. Filter-based method assumes that the energy of useful information in ECG concentrate in some specific frequency ranges. Then the noise can be removed once the ranges are estimated. Empirical mode decomposition decomposes signals into several intrinsic mode functions in a descending order according to frequency. Then baseline interference and high-frequency noise can be gotten rid of by removing low- and high-frequency parts. By applying the WT, ECG signals can be decomposed to the approximate (low-frequency component) and detailed (high-frequency component) information. Then thresholding is used in wavelet domain to smooth out or remove some coefficients of WT sub-signals of the measured signal. These methods process different segments with the same procedure, but in real conditions, no ECG beat is perfectly identical to another; rather, it may vary in terms of both morphology and duration [1]. The same procedure that is effective on one segment may not work on another.

In recent years, some scholars [12, 13] proposed denoising based on sparse decomposition. Every segment of signal will be decomposed into sparse parts and residues. Then the sparse parts are used to estimate pure signals, when useful information in the signals is assumed to be sparse. For each segment, the decomposition is implemented by using optimisation method to find the sparsest representation, so sparse-based method is non-linear. Since sparse-based denosing method is proved to be less risky [14], we use it for ECG signals.

## 2 Material and methods

### 2.1 Material

The experiment's data is from MIT-BIH arrhythmia database [15], which contains 48 records and each of it lasts 30 min. These records all contain two leads’ data: the modified lead II and one of the modified leads V1, V2, V4 or V5. The sampling frequency is 360 Hz. The given data have already been filtered by a band-pass filter at 0.1–100 Hz and the resolution is 200 samples per mV. In this Letter, the data from different records are segmented to 3 s, and a total of 40,790 segments are chosen randomly to test the results.

### 2.2 Sparse decomposition

The signal with noises can be modelled as

(1) $$y = x + sn\eqno\lpar 1\rpar $$

In which, *x* refers to the actual ECG signals, *sn* refers to noises and *y* refers to the signals observed. Denoising actually means estimating *x* in *y* and drawing *x* near to *y* as much as possible. The denoising based on sparse decomposition supposes the ECG signal *x* is sparse. Consequently, if having constructed an sparse dictionary *D*, we can find a sparse signal $x = D\hat \alpha $ that approximate the observed signal most and consider it as the original ECG signal's estimation, so as to find the minimum value of *L* as

(2) $$L = \displaystyle{1 \over 2}\mathop {\left\Vert {y - D{\bi \alpha }} \right\Vert }\nolimits_2^2 + \mu \mathop {\left\Vert {\bi \alpha } \right\Vert }\nolimits_0 \eqno\lpar 2\rpar $$

In which, $\mu $ is a regularisation parameter, used to balance the sparseness and the signal errors. $\mathop {\left\Vert {\bi \alpha } \right\Vert }\nolimits_0 $ refers to vector ${\bi \alpha }$ ’s zero norm, which represents the number of non-zero values in vector ${\bi \alpha }$.

### 2.3 Solving method

When *D* is complete and known, (2) offers an optimal object that sets ${\bi \alpha }$ as an unknown parameter. So estimate *x* in (1) means

(3) $$\hat \alpha = \mathop {\arg \min }\limits_\alpha \mathop {\left\Vert {\bi \alpha } \right\Vert }\nolimits_0 \quad s.t.\; \mathop {\left\Vert {y - D{\bi \alpha }} \right\Vert }\nolimits_2 \lt \Lambda \comma \; \quad x = D\hat \alpha \eqno\lpar 3\rpar $$

where $\mathop {\left\Vert \cdot \right\Vert }\nolimits_2 $ refers to two norms of a vector. Looking for the sparsest representation of a signal in a dictionary is a non-deterministic polynomial (NP) problem. There are two ways to solve the problem. The first one is the greedy pursuit algorithms [16], which approach the signals via an iterative process and choose a local optimal solution in each iterative process to reduce the errors gradually between the approximation signals and the original signals. These algorithms include matching pursuit algorithm and orthogonal matching pursuit algorithm [17]. The other typical algorithms are convex relaxation. The idea is to transform solving the NP problems in (1) into solving a linear programming problem. The typical solving method is basic pursuit [18]. In this Letter, we adopt the matching pursuit algorithm for sparse representation, for it is simple and effective.

When *D* is known, (3) offers an optimisation objective that sets ${\bi \alpha }$ as an unknown parameter, which can be used to estimate *x*. By using matching pursuit algorithm, ${\bi \alpha }$ can be solved iteratively. In the first step, *y* can be represented as

(4) $$y = \left\langle {y\comma \; d_i} \right\rangle d_i + Ry\eqno\lpar 4\rpar $$

To get a most sparse solution. It is necessary to minimise $\mathop {\left\Vert {Ry} \right\Vert }\nolimits^2 $ which means to find $d_i$ through (5)

(5) $$\left\langle {y\comma \; d_i} \right\rangle \ge \mathop {\max }\limits_{d \in D} \left\vert {\left\langle {y\comma \; d} \right\rangle } \right\vert \eqno\lpar 5\rpar $$

where $w_i = \left\langle {y\comma \; d_i} \right\rangle $ is a coefficient of atom $d_i$, $\left\langle {y\comma \; d_i} \right\rangle $ means product of *y* and $d_i$, *Ry* means residues.

Following that, *y* is replaced by *Ry*, and other $d_j$ and their coefficients could be found iteratively. Finally, (6) is obtained as follows:

(6) $$y = \sum {w_id_i} \eqno\lpar 6\rpar $$

As $w_i$ is calculated through iteration by using basis pursuit method, $\mathop {\left\Vert {y - \sum {w_i} d_i} \right\Vert }\nolimits^2 $ keeps decreasing while $\mathop {\left\Vert {w_i} \right\Vert }\nolimits_0 $ keeps increasing, we can get $\alpha $ as

(7) $$\alpha _i = \left\{{\eqalign{ & {w_i - \lambda } \quad {w_i \ge \lambda } \cr & {0} \quad \quad \quad \; \, { - \lambda \lt w_i \lt \lambda } \cr & {w_i + \lambda } \quad {w_i \le - \lambda } \cr } } \right.\eqno\lpar 7\rpar $$

where $\lambda = \sigma \sqrt {2\; {\log }_eN} $, where $\sigma $ is covariance of *y*, and *N* denotes the length of *y*. Since $w_i$ of different segments of signals vary from others, the sparse-based denosing is non-linear and can minimise the denosing risk [14].

### 2.4 Dictionary

The selection of sparse dictionary can directly affect the filtering effect. Naturally, the other core problem of denoising based on sparse-decomposition is how to construct a dictionary that suits a certain type of signals. In [19], Fourier coefficient and wavelet coefficient were presented as smooth signals’ sparse transform bases. Peyre [20] put forward several dictionaries composed of orthogonal bases, which can automatically search for a method to approximate a certain signal's optimal orthogonal base.

The sparse-decomposition under redundant dictionary is a process of finding the best linear combination composed of K-type of atom to represent a signal.

A relatively good filtering effect indeed can be obtained by using a large redundant dictionary, but it will inevitably increase the amount of calculation. In this Letter we investigate denoising result on several typical dictionaries in terms of symmetry, anti-symmetry and similarities of ECG signals.

Four dictionaries are investigated, namely the discrete cosine orthogonal base (with redundancy $r = 1$, as calculated by (8)), wavelet redundant dictionary (with redundancy $r = 3$ and includes the orthogonal wavelets composed by DB4, DB6 and DB8), Fourier dictionary and Gabor-based dictionary (with $k = 3$,whose mother function can be seen as in (9))

(8) $$d_k\lpar n\rpar = \left\{{\eqalign{ & {\displaystyle{1 \over {\sqrt N }}\comma \; } \quad \quad \quad \quad \quad \quad \quad \quad \quad \; \; \; {k = 0} \cr & {\sqrt {\displaystyle{2 \over N}} \cos \left({\displaystyle{\pi \over N}\left({n + \displaystyle{1 \over 2}} \right)k} \right)\comma \; } \quad {k = 1\comma \; 2\comma \; \ldots \comma \; N - 1} \cr } } \right.\eqno\lpar 8\rpar $$

(9) $$d_k\lpar n\rpar = \matrix{ {{\rm e}^{\sin \lpar kn\pi \rpar }} & {0 \le k \lt N} \cr } \eqno\lpar 9\rpar $$

### 2.5 Result evaluation

To evaluate the denoising effects and the signal distortion, signal–noise ratio (SNR, unit: dB) and minimum mean square error (MSE) between estimated signal and original signal are chosen. SNR and MSE can be expressed as

(10) $${\rm SNR} = 10 \times \mathop {\log }\nolimits_{10} \left[{\displaystyle{{\sum\nolimits_{i = 1}^N {Y_{\lpar i\rpar }^2 } } \over {\sum\nolimits_{i = 1}^N {\mathop {\lpar X_{\lpar i\rpar } - Y_{\lpar i\rpar }\rpar }\nolimits^2 } }}} \right]\eqno\lpar 10\rpar $$

and

(11) $${\rm MSE} = \displaystyle{1 \over N}\sum\limits_{i = 1}^N {\mathop {\lpar X_{\lpar i\rpar } - Y_{\lpar i\rpar }\rpar }\nolimits^2 } \eqno\lpar 11\rpar $$

$Y_{\lpar i\rpar }^2 $ is the original signal, $X_{\lpar i\rpar }$ is the estimated signal.

## 3 Results

WT can analyse signals in timeline and scale and has strong time–frequency localisation ability. As a result, it is one of the most efficient techniques for signal noise elimination, especially for non-stationary signal processing [21], and has been widely used in denoising ECG signals. So, in this Letter, WT-based denoising method is chosen as comparison to test the ECG signals from MIT-BIH arrhythmia database.

**Fig. 1:**
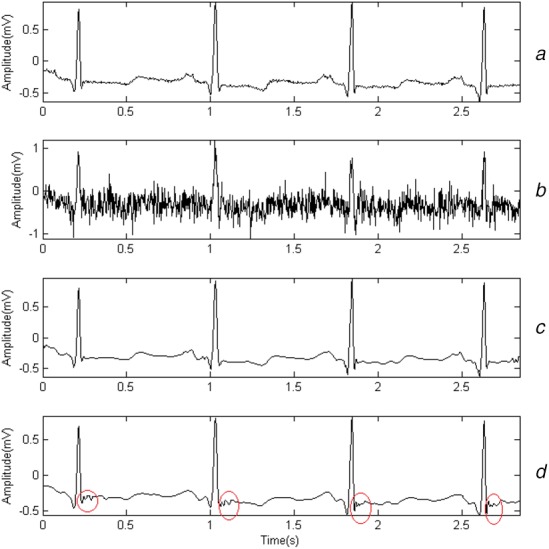
Denoising effect when amplitude of noises is 0.2 mV *a* Original signals *b* Noisy signal *c* Sparse-based method denoised signal *d* Wavelet denoised signal

**Fig. 2:**
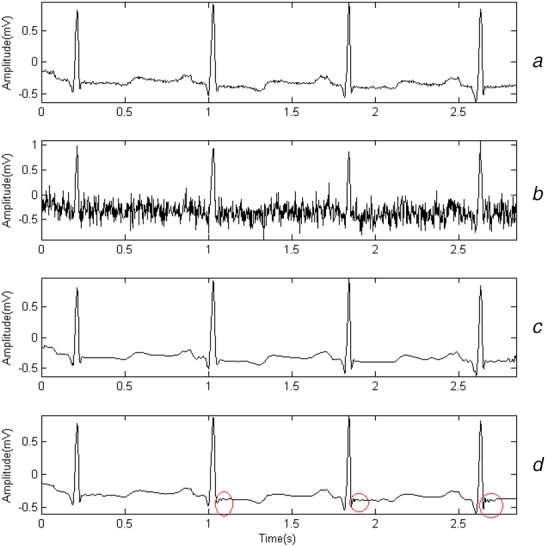
Denoising effect when amplitude of noises is 0.15 mV *a* Original signals *b* Noisy signal *c* Sparse-based method denoised signal *d* Wavelet denoised signal

**Fig. 3:**
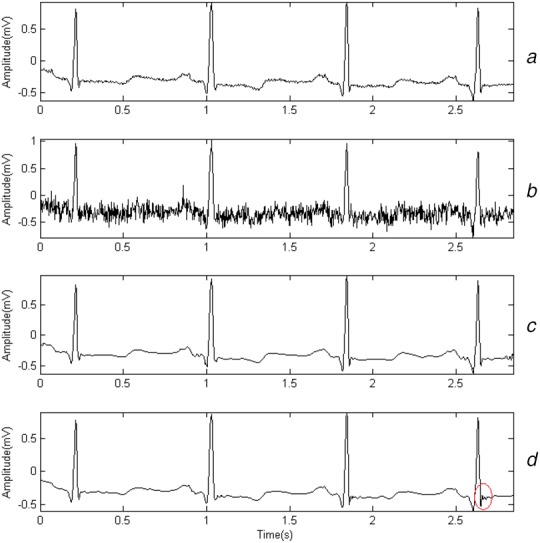
Denoising effect when amplitude of noises is 0.1 mV *a* Original signals *b* Noisy signal *c* Sparse-based method denoised signal *d* Wavelet denoised signal

Three kinds of mother wavelets that are similar to QRS in shape are chosen: db6, sym4 and bior3.3. Gaussian noises, whose amplitudes are 0.1, 0.15 and 0.2 mV, are added to simulate myoelectric interference. The denoising effects for a random segment from record 101 are shown in Figs. 1–3. Fig. 1*a* presents the original signals. Fig. 1*b* shows the noisy signal whose amplitude is set to 0.2 mV. Fig. 1*c* shows the result denoised by WT when db6 is chosen and Fig. 1*d* shows the result denoised by sparse-based method. The total duration of the segment is 3 s. Figs. 2 and 3 show the result in a similar way, except that the amplitudes of noises are 0.15 and 0.1 mV separately.

Table 1 lists the results of different methods when Gaussian noises with different amplitudes are added. The results are given by using SNR and MSE as indicators, which are calculated by (10) and (11). 40,790 segments which last 3 s are randomly selected from MIT-BIH arrhythmia database to test the present method. The average MSE and SNR are listed in Table 1. D1 is a dictionary composed of cosine and D2 is a dictionary composed of Db wavelets. D3 is a Fourier dictionary and D4 is a Gabor sine-modulated dictionary which is described in Section 2.4.

**Table 1:** Statistical of denoising results

Amplitude of input noise, mV	Method	Dic/thr	MSE	SNR, dB
0.2	sparse	D1	0.000858219	66.1551869
		D2	0.000618651	61.81878968
		D3	0.000223051	63.71338863
		D4	0.000882012	61.00150392
	wavelet	soft	0.001340884	55.95714751
		hard	0.007401719	60.57514088
0.15	sparse	D1	0.000609168	70.87557162
		D2	0.000341154	67.95521504
		D3	0.000134768	74.04089562
		D4	0.000808009	61.33545074
	wavelet	soft	0.001320988	60.30327266
		hard	0.010714962	60.87565888
0.1	sparse	D1	0.001196152	81.11762022
		D2	0.000828772	81.15857579
		D3	0.000340703	86.86536025
		D4	0.00871955	81.71984611
	wavelet	soft	0.001401552	81.00536294
		hard	0.011109764	71.48312186

## 4 Discussion

Fig. 1 shows the results of both WT-based method and sparse-based method for record 101. By comparing Fig. 1*a* with Figs. 1*c*, 2*a* with Figs. 2*c*, 3*a* with Fig. 3*c*, we find that the S waves are quite different after being denoised. It may be caused by a bad choice of threshold value. Actually, it happens in other records. The finding matches the fact in [22] that WT-based denoising may suffer from aliasing. Fig. 1*d* shows that by using sparse-based method, the result can be improved.

Table 1 shows the denoise results, using SNR and MSE as performance measures. It can be seen that as the larger the amplitude of the noise, the smaller the SNR value for both WT-based method and sparse-based method. However, for different levels of noises, sparse-based method gets larger SNR and smaller MSE than those of WT-based method, which demonstrates that sparse-based method outperforms WT-based method.

Another finding is that the selection of dictionaries can affect the filtering effect. It shows that there is little difference between the values of SNR when using the present dictionaries. However, we can acquire the highest SNR value and the minimum MSE value by using dictionaries composed of symmetric functions that are similar to ECG signals. We can get the second best denoising effect by using dictionaries composed of unsymmetrical orthogonal basis that are similar to ECG signals. The dictionaries composed of cosine and non-sinusoidal signals perform poorly. Therefore, the function that has good symmetry and is similar to ECG signals is strongly suggested to be used to compose dictionaries.

## 5 Conclusion

This Letter studies the ECG signal denoising based on sparse decomposition. The ECG signals are decomposed into sparse parts, which are estimated as pure signals, and other parts. The sparse-decomposition is implemented by using matching pursuit algorithm. This Letter makes major contribution to proving that the sparse-decomposition can avoid aliasing caused by WT-based denoising and guarantee filtering effect.

It is also found that selection of dictionaries can affect the filtering effect. A dictionary constructed by a basis function that has a similar profile with ECG could improve the effect. However, whether the finding is true of other types of signals is yet to be examined.

## 6 Funding and declaration of interests

Conflict of interest: none declared.
